# Analysis of Forming Limits in Sheet Metal Forming with Pattern Recognition Methods. Part 2: Unsupervised Methodology and Application

**DOI:** 10.3390/ma11101892

**Published:** 2018-10-03

**Authors:** Christian Jaremenko, Emanuela Affronti, Andreas Maier, Marion Merklein

**Affiliations:** 1 Pattern Recognition Lab, Friedrich-Alexander-Universität Erlangen-Nürnberg Martensstr. 3, 91058 Erlangen, Germany; Andreas.Maier@fau.de; 2 Institute of Manufacturing Technology, Friedrich-Alexander-Universität Erlangen-Nürnberg Egerlandstr. 13, 91058 Erlangen, Germany; Marion.Merklein@fau.de

**Keywords:** forming limit curve, pattern recognition, sheet metal forming, machine learning

## Abstract

The forming limit curve (FLC) is used in finite element analysis (FEA) for the modeling of onset of sheet metal instability during forming. The FLC is usually evaluated by achieving forming measurements with optical measurement system during Nakajima tests. Current evaluation methods such as the standard method according to DIN EN ISO 12004-2 and time-dependent methods limit the evaluation range to a fraction of the available information and show weaknesses in the context of brittle materials that do not have a pronounced constriction phase. In order to meet these challenges, a supervised pattern recognition method was proposed, whose results depend on the quality of the expert annotations. In order to alleviate this dependence on experts, this study proposes an unsupervised classification approach that does not require expert annotations and allows a probabilistic evaluation of the onset of localized necking. For this purpose, the results of the Nakajima tests are examined with an optical measuring system and evaluated using an unsupervised classification method. In order to assess the quality of the results, a comparison is made with the time-dependent method proposed by Volk and Hora, as well as expert annotations, while validated with metallographic investigations. Two evaluation methods are presented, the deterministic FLC, which provides a lower and upper limit for the onset of necking, and a probabilistic FLC, which allows definition of failure quantiles. Both methods provide a necking range that shows good correlation with the expert opinion as well as the results of the time-dependent method and metallographic examinations.

## 1. Introduction

The forming range of sheet metal is assessed in terms of the forming limit curve (FLC), where the limits are typically defined by the major and minor strain pairs, with the onset of necking. In Europe, the FLC is evaluated according to DIN EN ISO 12004-2 [1], where stretch tests are carried out according to the Nakajima [2] and Marciniak [3] setups. In these setups, the sheet metal is clamped in a clamping unit and subsequently, a hemispherical or flat-shaped punch deforms the specimen until fracture. The evaluation method proposed in the ISO standard is based on the Bragard study of 1972 [4] and is referred to as the “cross-section method”. In this approach, the strain distribution of the material is evaluated just before failure. Here, sections are defined perpendicular to the crack initiation and the strain development is approximated with a second-order polynomial. Traditional strain measurement techniques were based on a comparison of the size of circular patterns, visible on the specimen surface, before and after the end of the forming procedure. However, such an approach only permits the evaluation of the last stage of the strain distribution, without taking the strain history into account. Nowadays, the deformation behavior is analyzed using digital image correlation techniques (DIC) and used for the determination of the FLC [5]. A stereo camera system measures the strain on the material’s surface during Nakajima or Marciniak tests, and enables their progression to be monitored. However, the standard still relies on the “cross-section” method. Additionally, modern lightweight materials such as high-strength steels and aluminum alloys are characterized by several local strain maxima and sudden occurrences of cracks without a necking phase. Consequently, second-order functions are ill-suited to approximate the strain distribution of such materials.

In order to address the limitations of the “cross-section” method, several techniques which take the forming history during Nakajima tests into account, have been proposed. These time-dependent methods include the so-called “line-fit” approach proposed by Volk et al. [6] and the correlation coefficient method [7]. Both take into account the reduction in thickness observed, within a pre-defined zone of instability, and determine the onset of the necking based on sudden changes in the thickness of the specimen. One disadvantage of these time-dependent methods is their prerequisite for the zone of instability to be defined, which limits their focus and adversely affects the overall evaluation.

In 2015 [8] a machine learning-based approach was investigated, to introduce new insights into forming development. Conventional pattern recognition deals with the automatic processing and evaluation of data [9], whereby a physical signal, e.g., images or speech, is first converted into suitable compact characteristic features. Typically, characteristic features are chosen in a way that reflect the visual perceptions of experts who have a strong understanding of the problem to be solved. To enable an automatic separation into classes, experts assign different labels to the data. Based on a classification algorithm and a representative subset of the data, decision boundaries are learned using the feature representation/label pairs, such that the hypothesis of the learned class boundaries can be verified using the remaining disjoint data. In this first approach in 2015, a pattern recognition process was used to predict the crack class of a DX54D steel during the forming process, which achieved a prediction accuracy of 90% before the actual cracking was observed. Metallographic investigations have shown that both, conventional deep-drawing steels [10] and dual-phase steels [11], show patterns associated with localized necking on the surface, which enables the use of pattern recognition to determine forming limits [12]. In [13] (referred to as Part 1 in the following) this idea was extended by assessing multiple evaluation areas and comparing the obtained classification results with experts, for multiple failure classes (diffuse, local constriction and crack). It was demonstrated that expert knowledge can be used to determine the localized necking consistently. However, the accuracy and efficacy of results obtained, are substantially influenced by the subjective nature of the experts’ annotations, in addition to external factors such as the sampling frequency, especially in case of the diffuse necking class. Additionally, an expert assisted evaluation of material failure is time-consuming, expensive and therefore reduces applicability.

To address these issues, this study focuses on the localized necking and presents an unsupervised classification approach based on One-Class Support Vector Machines (SVM) [14,15], which does not require expert knowledge and enables a probabilistic assessment of localized necking, using objective gradient information. The same data set of Part 1 is used for this purpose, wherein the time derivative is used to emphasize the progression of strain across successive images, as suggested by Vacher et al. [16]. The dataset consists of a deep-drawing steel DX54D in two different thicknesses, a dual-phase steel DP800 and an aluminum alloy AC170. The results of the unsupervised pattern recognition approach are compared with the annotations of the experts and the results of the time-dependent FLC, with the main focus on time-accurate determination of the onset of localized necking.

## 2. Experimental Procedure and Materials

**Figure 1:**
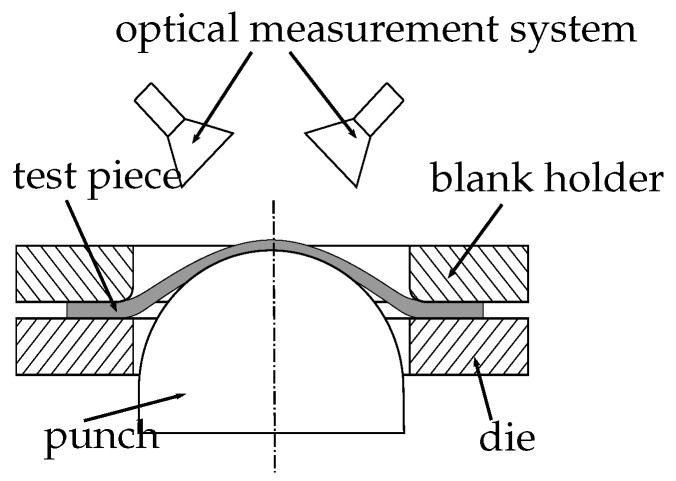
Schematic of the Nakajima experimental setup.

The FLC can be experimentally determined using a Nakajima test setup (self-developed testing machine at the Institute of Manufacturing Technology, Erlangen, Germany). The system consists of a stereo camera system, a clamping unit with an inner die diameter of 110 mm, and a hemispherical punch with a diameter of 100 mm (cf. Figure 1). An optical measurement system (ARAMIS gom GmbH, Braunschweig, Germany) is used to record the forming process and to calculate the strain distributions using DIC techniques. Specimen are prepared with a white primer and a stochastic pattern of black graphite to enable image correlation. A lubrication system according to DIN EN ISO-12004-2 is used to minimize the friction between punch and specimen and to induce a rupture at the top of the specimen. By changing the specimen geometry, different strain paths are obtained, from a full geometry to tapered blanks with parallel connections, where the width of the remaining material determines the name of the geometry (e.g., S050 corresponds to 50 mm). Punch velocity and sampling rate are varied from 1 to 2 mm/s and 15 Hz, 20 Hz or 40 Hz, respectively, to provide different combinations of strain paths, materials, and boundary conditions.

Three different materials are evaluated within this study: (1) DX54D, a deep-drawing steel, with ductile necking behavior and observable localization on the surface; (2) DP800, a dual-phase steel, high-strength material with a matrix of ferrite and martensite precipitations and hence multiple observable local maxima during Nakajima tests; (3) AC170, a lightweight aluminum alloy of the 6xxx series with multiple maxima during Nakajima tests under plain strain conditions. The principal material properties are summarized in Table 1. For a comprehensive description of the forming behavior of the evaluated materials please refer to Part 1.

## 3. Method

The proposed unsupervised classification approach follows a typical pipeline used in pattern recognition algorithms, which consists of four sequential steps: data acquisition, preprocessing, feature extraction, and classification. In the classification step, the data is divided into a disjoint training and test set. Within supervised classification, the training set coupled with the ground truth class label vector, is used to learn a decision boundary to optimally separate the class members within the training set from each other. The unseen test data is used to simulate a real-world scenario and to assess the quality of the learned decision boundary. In this study, an unsupervised classification approach based on One-Class-SVM [14,15] was employed, which can be trained without the need for expert annotations or ground truth labels. The previous study showed, that gradient-based features, such as Histogram of Oriented Gradients (HoG) [17], were well-suited to identifying localized necking in a supervised manner. They achieved the best performance when used together with the extraction of multiple patches (9) from the sample images. Additionally, it was shown that the experts annotated the data based on sudden changes in the cross-sections through strain distributions. Therefore, this study uses the time derivative of video sequences, which highlight differences between successive frames. As only small parts of the image information change from one frame to the other, the evaluation area must be sufficiently large to cover the necking region.

### 3.1. Preprocessing

The data used in this study was acquired using the ARAMIS optical measuring system (v6.3.0-7), and is in the form of a three-channel video sequence comprising, major strain, minor strain and thinning. These sequences may contain defect pixels, due to specimen preparation or DIC failure, which adversely affect the time derivative of the video sequences. Therefore, where possible, temporal information of the strain progression of single pixels is used to interpolate missing values. If no information is available for single pixels across the entire forming procedure, the average strain value of a 3×3 neighborhood is used to replace the defect pixel. Additionally, as the video sequences included a varying number of frames/images, with a multitude of defect pixels used to identify crack initiation, the sequences were cropped and restricted in length, automatically. As the presence of defect pixels is indicative of the onset of material failure, they are used to shorten the video sequences and ensure that feature extraction and subsequent analyses are conducted using valid and descriptive image information. After defect pixel interpolation, the time derivative of the video sequences is estimated using backward difference approximation. Hence, only the differences between successive images are emphasized, serving as inputs to the algorithm. Additionally, to reduce the influence of measurement errors, e.g., single pixels that are off by a large magnitude, a quantile normalization scheme is applied on every individual image. This uses the 0.5% and the 99.5% percentile of the image intensities as lower and upper bounds, convert the intensity range to 0–1. Furthermore, the expected necking area is defined using the maximum strain on the last valid frame without defect pixels, and subdivided into nine patches with a side length of 32 px.

### 3.2. Feature Extraction

HoG [17] is a feature descriptor which was originally introduced for the task of pedestrian detection. To derive the feature descriptor, the image is subdivided into small non-overlapping rectangular areas, called cells. Within each cell, the edge response is evaluated using the magnitude and the orientation, whereby the orientation defines the position of the histogram bin, in which the magnitudes are summed up. Multiple cells can be concatenated into a larger block for contrast normalization. As the evaluation area within this study focuses on the expected necking area, subdividing into small cells is omitted, as well as the contrast normalization, as the pixel intensities are calculated using an optical measurement system and hence no lightning differences occur. Additionally, the orientation resolution is set to 5° to support fine-grained sensing.

### 3.3. Classification Using One-Class-SVM

SVM [18] approximate a hyperplane, that separates instances of one class from another using the available training data and the corresponding ground truth label vector, by maximizing the margin between the individual boundaries. In an unsupervised setup, the ground truth labels are unknown, rendering the generation of a separation hyperplane difficult. One-Class SVM [14,15] solve this problem by estimation of compact regions in feature space, which contain a selected fraction of the training data. One can visualize the problem as finding the center and radius of a sphere [19], that covers most of the training data. These compact regions define the inlier class, and the remaining instances that lay outside the compact regions are considered as outliers. Hence, most of the unknown data distribution is covered, without the necessity to approximate it. The test set is used to verify the separation hypothesis and instances are considered as inliers they are within this sphere, and as outliers if they are elsewhere, whereby the confidence being classified as inlier decreases with increasing distance to the center. Two parameters (ν, γ) affect the shape and support of the One-Class SVM. The first parameter depicts the fraction of tolerated outliers in the training dataset and the second parameter defines the width of the Gaussian radial basis function kernel (RBF), that controls the influence and support of features. The One-Class SVM used in this study was trained using data solely from the homogeneous forming phase (2 mm punch movement), while the remaining data of unknown forming phase (homogeneous and inhomogeneous, 2 mm punch movement) was used as the test set. The ν parameter is naively set to 0.05, as only clean data, without necking behavior and with only a small amount of measurement errors, is expected. The γ parameter is naively set to 1/n, where *n* denotes the number of features per image. In general, the problem can be interpreted as anomaly detection, where an anomaly is defined as the deviation from the expected range of gradients that occur within the homogeneous forming phase.

#### 3.3.1. Deterministic FLC

A deterministic FLC is computed using the confidence scores output by the One-Class SVM. The scores are binarized, such that an instance can either be an inlier or an outlier independent of the level of confidence. Nine patches are extracted from each image, whereby each patch is classified independently. Since it is impossible to prove if the onset of necking within a specimen is determined by the classification of a single patch as an outlier, or if it is necessary to have all nine patches classified as outliers, two FLC candidates are introduced. The first candidate (SVMe) determines the last valid frame of each sequence of the test set that does not include any patches classified as outliers and uses this point in time to look up the strain value pairs. The second candidate (SVMf) determines the first frame of each sequence of the test set with all patches being classified as outliers. Thus, they can be considered as the onset and end of the localization phase. Especially in case of the SVMe candidate, single patches being erroneously classified as an outlier is inevitable, due to the presence of measurement errors. Therefore, the first stage without any patch classified as an outlier of each sequence is found by parsing through the video sequences backward starting from the last image of each sequence.

#### 3.3.2. Probabilistic FLC

**Figure 2:**
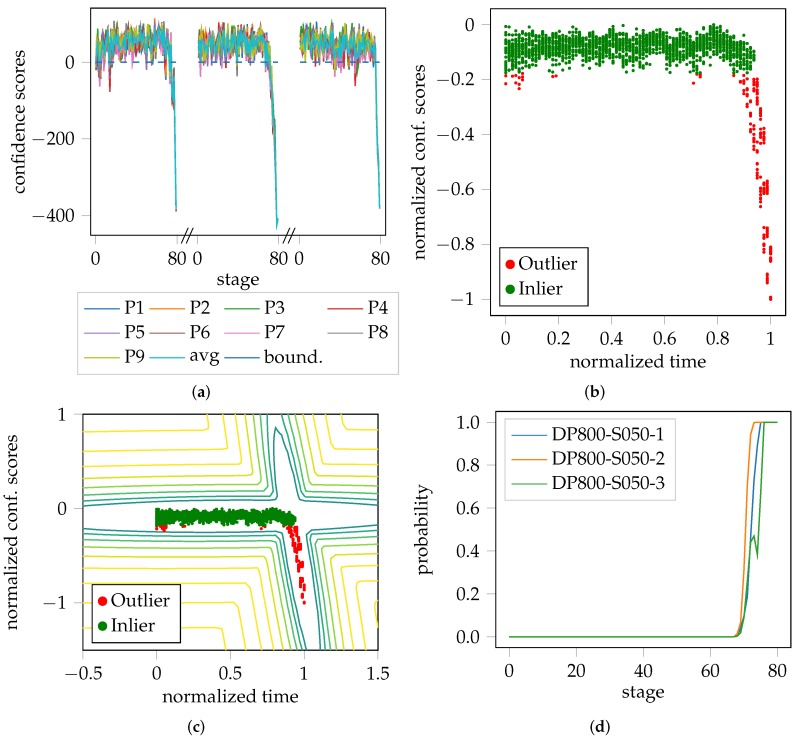
Exemplary procedure of the anomaly detection pipeline based on DP800-S050: (**a**) Confidence scores per patch including the average of the patches of each of the three trials; (**b**) Combined confidence scores of the previous trials with normalized time and scores; (**c**) Confidence scores with negative log. likelihood space of the modeled GMM; (**d**) Average probability of being an outlier over time and its progression for each individual trial of DP800-S050.

The probabilistic FLC proposed in this study, is a post-processing strategy that takes advantage of the fact that the video sequences in the test sets are pre-processed and shortened to have the same length (in time). Consequently, time can be incorporated as a feature/variable and used as an additional source of information. In general, the confidence scores of the One-Class SVM are unbounded, since they represent distances. As the test set covers the remaining homogeneous and the complete inhomogeneous part of the forming process of each trial, the minimum and maximum values can be used to normalize the confidence scores to fall within the range 0–1. The fact that the trials per test set share the same length and duration of time, allows them to be combined into one distribution as a function of normalized confidence scores over time. This distribution comprises of two parts, the homogeneous forming phase, and the inhomogeneous forming phase. A Gaussian mixture model (GMM) with two Gaussians are used to approximate the distribution of the data with the expectation-maximization algorithm (EM) [20]. The GMM centroids were initialized with the mean of the inlier and outlier classes. This way, it is possible to assign probabilities to the individual patches, while the average of nine patches per image is used to calculate the average probability of each image in the test sequence. The whole evaluation pipeline is visualized in Figure 2, starting with the unbounded confidence scores Figure 2a estimated per patch, for the three trials of one geometry. Towards the end of each trial, the decreasing confidence is a sign of the onset of necking, although the samples are still considered to lie within the inlier class/homogeneous forming phase. This is better emphasized in the combined normalized distribution of Figure 2b. Additionally, one can find outliers at the beginning of the distribution, and towards the end, which are most likely caused by temporal measurement noise. Figure 2c visualizes the negative log. likelihood space of the GMM with two Gaussian distributions. Clearly, the outliers at the start and end of the sequence are now considered to be part of the inlier class/distribution. The average probability per image of each trial of the test set is depicted in Figure 2d, wherein the probability of belonging to the outlier class rises towards the end of each trial.

**Figure 3:**
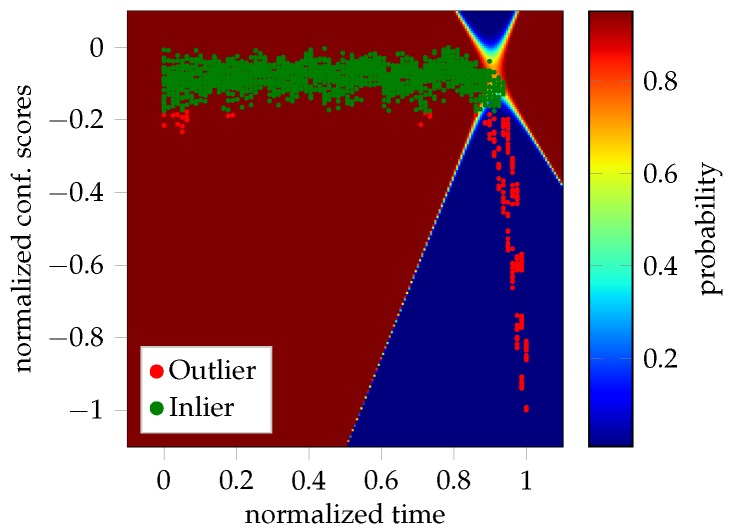
Decision boundaries of the GMM. The transition of belonging to the inlier class is emphasized by the color-coded probability in the curved region.

As mentioned, the onset of necking is expected to occur in the elbow region of the curves depicted in Figure 2, while still being considered as inlier. This is visualized by Figure 3, where the color-coded probability helps to depict the transition from the homogeneous to the inhomogeneous forming phase.

### 3.4. Experiments

The determination of the forming limit can be interpreted as an anomaly detection problem, as the main part of the data corresponds to the homogeneous forming phase, while the data of the inhomogeneous forming phase is usually underrepresented. In the “line-fit” method, the last 4 mm of the punch displacement are analyzed. The strain distribution is divided in stable condition, namely from 4 mm up to 2 mm before crack, and in instable condition after the onset of necking in the last 2 mm of punch displacement [21]. In the present investigation, in order to make a comparison with the “line-fit” method, the dataset is limited to the last 4 mm of the forming process. The first 2 mm of the sequences, describing the homogeneous phase, is used to train the One-Class-SVM. The last 2 mm is used for the detection of the anomaly. In principle the amount of data used for training the classifier and fitting the Gaussian can be chosen arbitrarily. The only constraint is that data from the homogeneous forming phase must be included. For this reason, the data set for DX54D (2.00 mm) deviates from the 4 mm restriction (extended to 6 mm), while still preserving the 50% training and test split. The geometries available for each material, their dataset size and the process parameters are summarized in Table 2.

**Figure 4:**
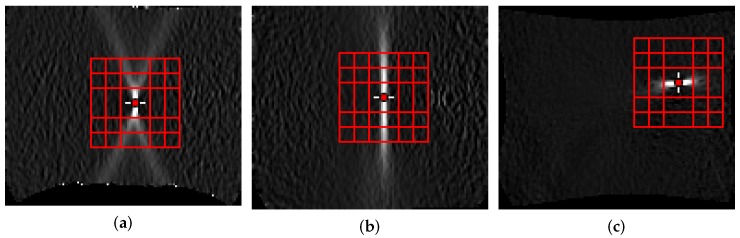
Multiple patches of the region of interest of different geometries: (**a**) DP800-S050-3 uniaxial; (**b**) DP800-S110-2 plane strain; (**c**) DP800-S245-1 biaxial.

For each material, every geometry was evaluated separately, individually. The first 2 mm of each of the three trials per geometry were combined into one data set and used to train the classifier. The combination of multiple trials enables estimation of an average forming limit curve (FLC) per geometry, which is of particular interest in most applications. As mentioned previously, the size of the evaluation area must be chosen such that, the area of interest is included independent of the underlying geometry, and hence the patch size was set to a side length of 32 px. Every patch contains the localizing effect, and the strain development over time with small variations, as the patches are shifted with a step length of 8 px. An example of the patch selection strategy used is depicted in Figure 4 for multiple geometries. Augmentation is used to further increase the size and variation of the training data set. This means the images are randomly rotated (2–15°) and flipped (horizontal, vertical, both) to address the different possible orientations of specimen. In case of e.g., DP800 this leads to a training data set of 3×80×12×9=25,920 patches. Quantitative evaluation of the method is difficult, as it would require ground truth based on metallographic examination of the exact point in time when necking occurs. To assess the quality of the method, the results are qualitatively compared with the related time-dependent “line-fit” method and with the experts’ annotations from Part 1.

## 4. Results and Discussion

### 4.1. Deterministic FLC

**Figure 5:**
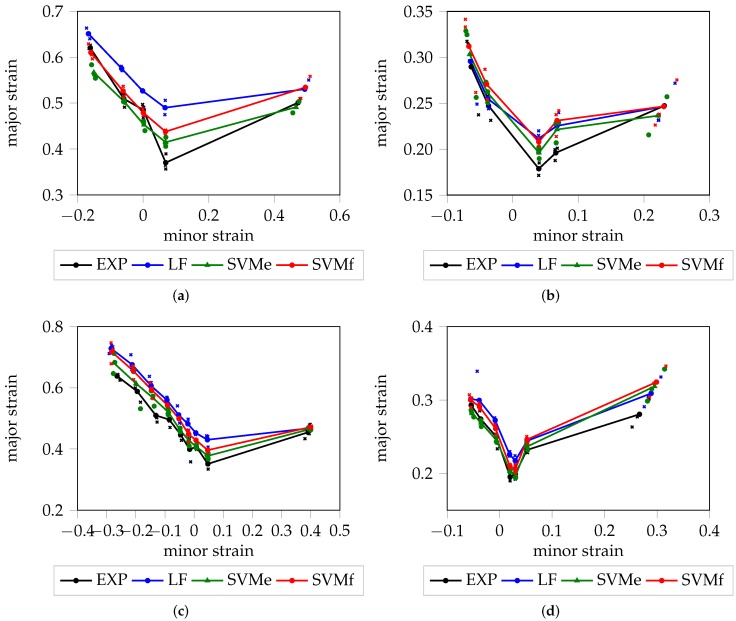
Deterministic FLC in relation to the “line-fit” method and experts’ decisions: (**a**) DX54D (2.00 mm); (**b**) DP800; (**c**) DX54D (0.75 mm); (**d**) AC170.

A graphical representation of the deterministic FLC for each material is visualized in Figure 5. Within all materials with the exception of DX54D (2.00 mm), SVMf shows high consistency with the “line-fit” method, which is reasonable as both strategies evaluate comparable information domains and make use of the difference between consecutive images. As expected, the SVMe estimate is consistently below that of SVMf, for all materials, as an earlier point in time is used to look up the strain value pairs. Especially in case of DP800, the three candidates, SVMe, SVMf and “line-fit”, are close to each under uniaxial loading conditions. For DX54D (2.00 mm), the rather large gap between the “line-fit” method and the SVM results may be a consequence of the rather low sampling frequency, or due to the different evaluation areas used to determine the onset of necking. There is high agreement between the SVMe results and the experts’ annotations from Part 1, with the latter being marginally below the former, for all materials with the exception of the uniaxial loading conditions of DX54D (2.00 mm). This means, that the experts consistently decided to select an earlier stage for the onset of necking based on the original strain distributions. This seems reasonable as the experts were looking for early sudden increases the strain distribution from one stage to the other.

### 4.2. Probabilistic FLC

**Figure 6:**
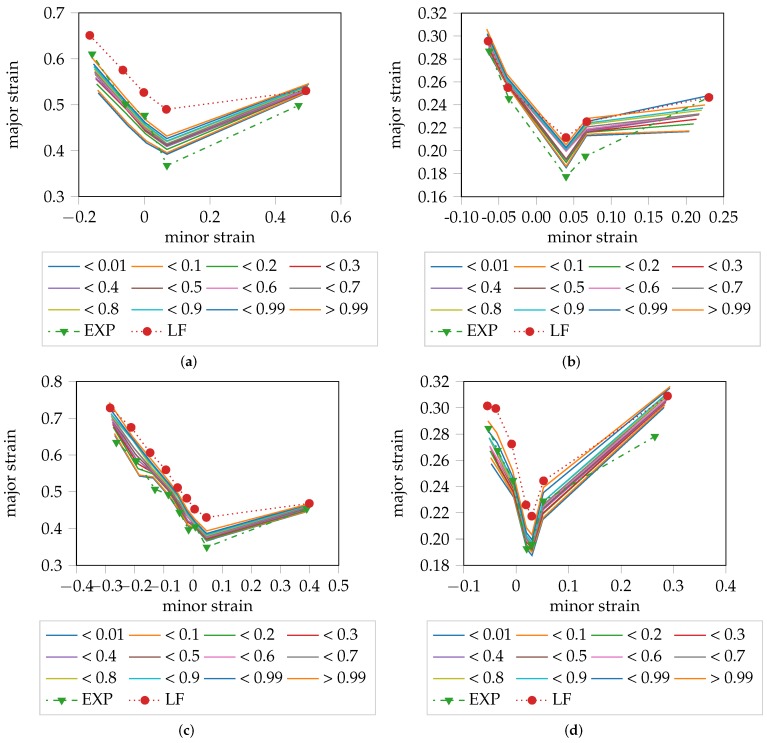
Probabilistic FLC in relation to the “line-fit” method and experts’ decisions: (**a**) DX54D (2.00 mm); (**b**) DP800; (**c**) DX54D (0.75 mm); (**d**) AC170.

Overall, the experts’ annotation lay below the SVMe results, leaving room for improvement in finding the point in time when the onset of necking begins. Taking into consideration that the experts’ annotations contain some source of error in defining the onset of necking due to variance between the experts or a high sampling frequency, as depicted by Figure 3, the confidence of being an inlier decreases towards the end of the forming process, before being classified as outlier. This decrease is expressed in terms of quantiles of probabilities as belonging to the outlier class. The probabilistic FLC for each material is visualized in Figure 6. To be able to assess the quality of the result, the “line-fit” method as well as the experts’ annotations are depicted for comparison. In the case of DX54D (0.75 mm), excellent agreement between the estimated probabilistic FLC, of the category < 0.01 quantile, and the experts’ annotations was observed over all geometries. This was only true for the uniaxial loading conditions in the case of AC170 and DP800. DX54D (2.00 mm) shows the largest deviation from the <0.01 quantile, which might once again be the result of the low sampling rate combined with the ductility of the material. The deviation from the plane strain to biaxial strain conditions on the right side of the curve of AC170 and DP800, are a result of the poor quality of the experts’ annotations, since in these cases the onset of necking could not be defined in consistent manner. As expected, the >0.99 quantile shows very good agreement with the “line-fit” result, in all cases except DX54D (2.00 mm). The remaining quantiles fall between the <0.01 quantile and the >0.99 quantile.

### 4.3. Comparison with Time-Dependent Evaluation Method

The “line-fit” method evaluates comparable information to the proposed approach to determine the onset of localized necking, and consequently, is a suitable choice for comparison. The main difference is in the definition of the evaluation area for the former, as the first derivative in the thinning direction is used to determine the evaluation area with an additional restriction to 15–20 connected pixels. This area is averaged for every stage of the forming process, and the resulting curve is used to determine the onset of necking based on the intersection of two lines that are regressed in the homogeneous and inhomogeneous area of the curve. Consequently, choice of the evaluation area is crucial for the “line-fit” method as it directly affects the shape and steepness of the curve. In contrast, the approach proposed in this study identifies a certain point in time when something abnormal starts to take place with respect to the homogeneous forming area, e.g., a rising gradient, meaning that with a certain confidence at time x, the likelihood of being in the necking phase rises with progression of the forming process. The choice of pixels that are used to determine the forming limit can be chosen independently, as long as the necking area with rising gradient is included in the evaluated patches. In order to facilitate comparisons between the proposed approach and the line fit method, a threshold value of 0.9 of the maximum thinning ($\phi_{3}$) was used, with additional restriction to 15–20 connected pixels. The proposed method is independent from the evaluation area, as it returns a point in time to look up the strain value pairs, and consequently the evaluation area could be defined using other constraints.

**Figure 7:**
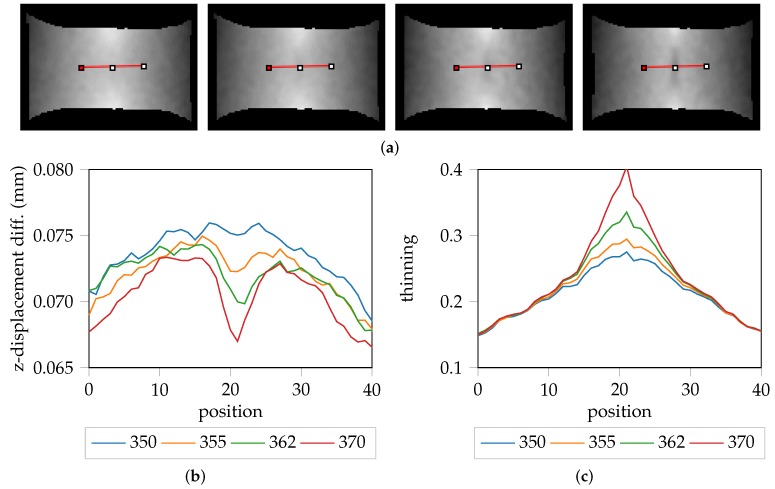
Evaluation of the z-displacement progression over time of DX54D (2.00 mm)-S030-1: (**a**) Visualization of the z-displacements of subsequent stages, with cross-sections at stage 350,355, 362 and 370; (**b**) Visualization of the corresponding cross-sections as profiles; (**c**) Corresponding cross-section profiles of the original strain distribution.

The hypothesis that the “line-fit” method overestimates the onset of localized necking is supported with the evaluation of the z-displacement differences of DX54D (2.00 mm)-S030-1 as visualized in Figure 7a. In this trial, the “line-fit” method returns stage 370 as the onset of necking. However, this seems to be optimistic as already some reduction of sheet metal thickness along the z-axis is already visible as shown in Figure 7a (right). Investigation of the earlier stages revealed that some reduction in sheet metal thickness was already present at stage 355. Consequently, the z-displacement of specimen is a valuable source of information that could be exploited when evaluating ductile materials. This is further emphasized by the z-displacement cross-section profiles depicted in Figure 7b. Additionally, Figure 7c depicts the corresponding cross-section profiles of the strain distribution.

**Figure 8:**
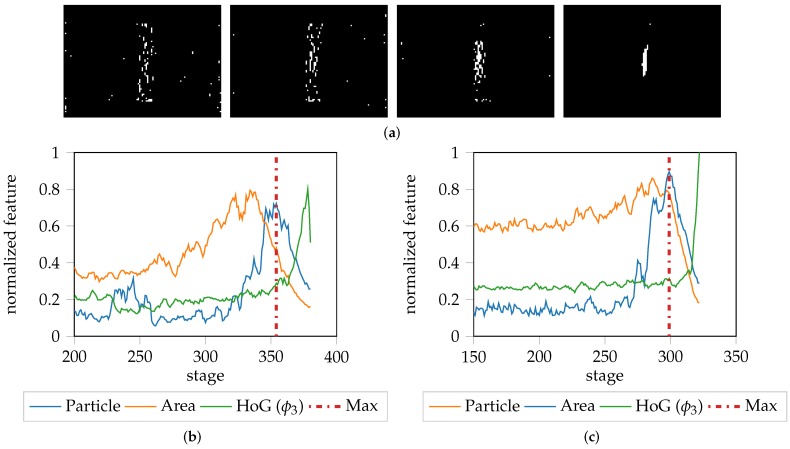
Evaluation of the thresholded area progression in relation to the HoG feature: (**a**) Concentration and increase of the thresholded area of DX54D (2.00 mm)-S030-1 of $\phi_{3}$ difference images at stage 300, 330, 360 and 370. First the area above the 0.9 threshold is spread over the whole image before concentrating to a single particle; (**b**) Progression of the size of the thresholded area of the whole image (orange) with respect to progression of the size of the largest connected particle (blue) and the progression of the HoG feature of DX54D (2.00 mm)-S030-1 (Green); (**c**) Corresponding information based on DP800-S050-1. After reaching the maximum possible connected particle size, no further forming is possible, leading to an increasing gradient in various HoG. bins, depending on the underlying geometry.

As already mentioned, the choice of evaluation area to determine the onset of necking is critical. At the beginning of the forming procedure, the thresholded area is distributed over the whole evaluation area and as the forming process progresses, this area gets concentrated towards the center, finally resulting in a single connected particle. This is depicted in Figure 8, which compares the change in area size (using a threshold of 0.9 on $\phi_{3}$ ), with the change in the size of the largest connected particle.

This process is depicted by Figure 8b, where the relationship between the size of the thresholded evaluation area and the largest connected particle, is emphasized. The thresholded area reaches its maximum before the single connected particle. Additionally, after the connected particle reaches its maximum, the HoG feature starts rising, which indicates that the necking area is unable to spread any further. This leads to an increasing gradient and emphasizes HoG is a reasonable choice as a feature descriptor. The aforementioned observation is dependent on the material properties, especially the ductility. However comparable characteristics of this effect can be found when evaluating different materials e.g., DP800-S050-1 as depicted by Figure 8c. The thresholded area maximum and connected component maximum show high correlation, and after some stages the HoG feature starts rising. This additionally shows the dependence on the threshold, as a different threshold e.g., 0.95 would most likely result in a maximum at a different point in time, particularly with regard to the connected component. This highlights the need to find a material dependent evaluation area rather than using a fixed size of threshold or amount of pixels to determine the onset of localized necking. For example, for DX54D (2.00 mm)-S030-1 from Figure 7 and Figure 8 an optimal evaluation area would include the complete necking area, which corresponds to a width of around 15 px, as defined in the cross-sections plot shown in Figure 7b. This was inferred based on the fact that at position ≈15 and ≈27, in the strain distribution plot shown in Figure 7c, the strain remains static. Since the dependence on a threshold always influences the evaluation area and thus the resulting onset of necking, it might be advantageous to find the necking area using image segmentation techniques. However, since the onset of necking in this study is found independently of the evaluation area, it would be possible to simply use the maximum strain value of the necking stage rather than averaging an approximated area.

**Figure 9:**
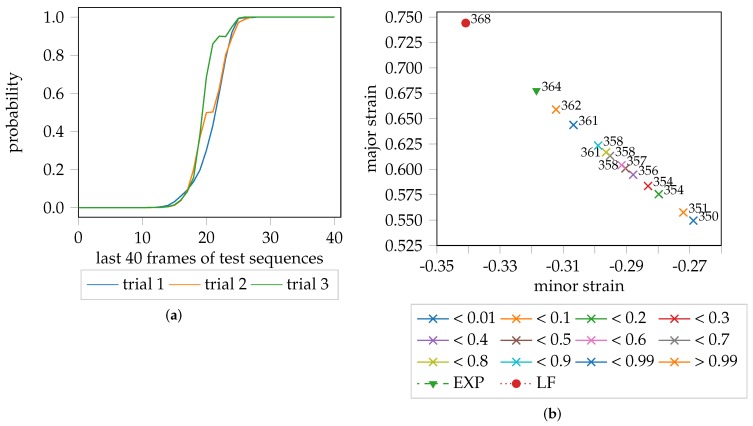
Probability progression and comparison of the averaged lookup time steps per quantile with the average “line-fit” time step lookup of DX54D (2.00 mm)-S030-1: (**a**) Probability progression per trial with normalized time; (**b**) Average major and minor strain of the quantiles with averaged lookup time information.

Leaving aside the actual evaluation area and hence the actual major and minor strain pairs, the point in time when necking is detected, can be compared. As the present study combines three trials for each geometry to determine the probabilistic FLC candidate, the average time over three trials is depicted in Figure 9b for each quantile. There is a probability of <1% that necking is initiated before stage 350. This increases to a 50% probability by stage 356 and is >99% at stage 362. The “line-fit” method for DX54D (2.00 mm)-S030-1 suggests stage 368 to be the onset of necking on average, whereas the majority of experts from Part 1 identified stage 364, which is reasonable as they concentrated on finding a sudden increase of strain from one image to the other based on cross-sections through the strain distributions. The multiple time points in Figure 9b are a result of the variation in the slopes of the probability progression curves, per trial, as depicted in Figure 9a. As the trials have different lengths, the image only contains the last 40 frames of the test set per video sequence and trial.

### 4.4. Comparison of Deterministic and Probabilistic FLC

**Figure 10:**
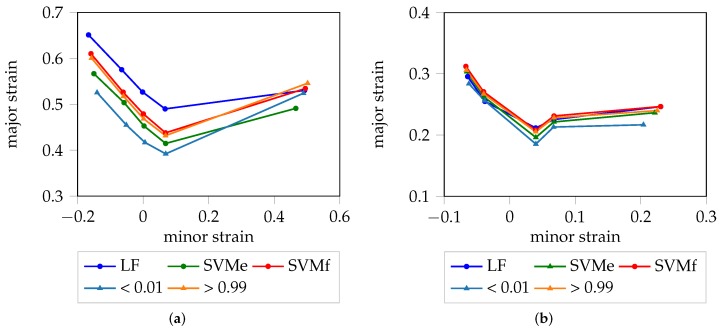
Comparison of D-FLC and P-FLC: (**a**) DX54D (2.00 mm); (**b**) DP800. Very good agreement between the SVMf and >0.99 quantile is reached. As expected, the <0.01 quantile lays below the SVMe curve. As the quantiles are based on the GMM in contrast to the SVMe, the S245 (right most side of each plot) of SVMe must not agree due to noise and instable outliers.

The deterministic and probabilistic FLC are both capable of describing the onset of necking. The D-FLC uses the binary affiliation of patches to the outlier class without consideration of the class-membership of neighboring patches. Within the P-FLC, two Gaussians are used to model the homogeneous and inhomogeneous classes and thus allow to extract probabilities for each patch and stage. This is of particular interest in the curved region, within the transition area from the inlier to the outlier class, as it enables detection of the onset of necking, based on the change in the gradient information. Furthermore, this detection is expressed in the form of a likelihood of necking, enabling a probabilistic interpretation of the forming process. For DX54D we found that the >0.99 quantile estimate of the probabilistic FLC was in close agreement with SVMf. For DP800, the line-fit method also showed strong consistency with these two approaches as depicted in Figure 10. Due to the different evaluation schemes, the <0.01 quantile was below the SVMe curve, as it is possible to evaluate the data, that is not yet recognized as outlier, but develops into this direction. While the difference is rather large in case of DX54D (2.00 mm), it vanishes in case of DP800 due to its lower ductility. The <0.01 quantile and SVMe must not generally agree as visualized in Figure 10a, as measurement noise or unstable outliers may affect the modeling of the distributions as well as the lookups of SVMe. Another major difference between these two evaluation strategies, is that the deterministic FLC might be extended into an on-line evaluation method, which is capable of stopping the forming process, when being trained in an incremental manner, e.g., to allow generation of ground truth based on metallographic examinations. So far, an on-line method would only be possible with the SVMf approach, as nine patches being classified as outliers is unlikely during the homogeneous forming phase. Such an on-line evaluation method is impossible in case of the probabilistic FLC as it exploits time information.

### 4.5. Comparison with Metallography

**Figure 11:**
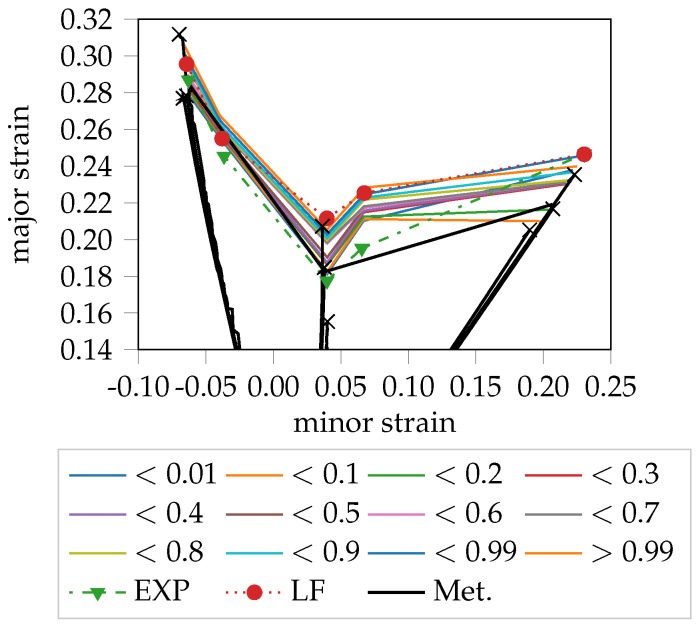
Probabilistic FLC of DP800 compared with the LF, EXP method and outcomes from the metallographic analysis presented in [11].

A quantification of the method can be achieved with the analysis on the material behavior using metallography. In [11], a metallographic analysis of a DP800 at several forming steps and different strain paths using Nakajima tests has been presented. This analysis of the surface and thickness pointed out that the onset of necking for this dual-phase steel starts on the surface with micro-cracks that are metallographic recognizable. The micro-crack corresponds to multiple localization in the strain distribution and thus this information can be used for the definition of the class “onset of instability” and serve as ground truth in supervised pattern recognition approaches. Once the forming step is selected, the corresponding strain level in terms of major and minor strain can be found. The outcomes for the different strain paths are compared in Figure 11 with introduced probabilistic FLC. It can be observed, that the forming level defined by the metallographic evaluation are in good agreement with the outcomes of the unsupervised method. In particular, the average FLC values from the metallographic analysis cover the expert evaluation, with the exception of the S245 geometry. Under biaxial straining, as already discussed in Part 1, the strain distribution is homogeneous on the surface most of the time and the main straining occurs in the thickness, due to the volume constancy. Therefore, the evaluation of onset of instability for this geometry is still challenging. Nevertheless, the experimental evaluation with metallography agree with the quantile distribution, confirming that the unsupervised evaluation of the onset of instability is plausible. The LF method gets in general higher FLC results than the metallography observations. According to the quantile investigation, the LF is at the same level of high quantile, namely >0.99. That means, using the LF method, the evaluated necking phase is in advanced development stage and therefore is less conservative. Instead, the experts are conservative and detect a very early stage of localization. In general, the comparison with the metallographic analysis confirms the qualification of the unsupervised method and gives an overview of the level of development of the necking using quantiles.

### 4.6. Factors of Influence

**Figure 12:**
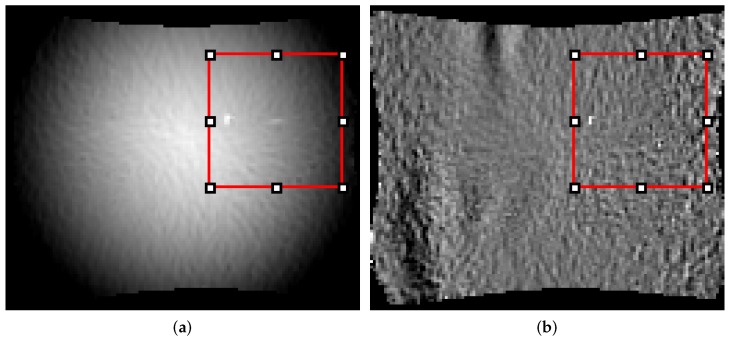
Visualization of measurement noise and its influence on the difference: (**a**) Original strain distribution with pixel artifacts; (**b**) Corresponding difference with pixel artifacts.

As already mentioned a couple of factors influence the quality of the result. One critical part is the preparation of the specimen, as it directly affects the DIC measurements. Sub-optimal preparation reduces the traceability of blocks and thus may lead to defect pixels or incorrect measurements that lead to alternating or fluctuating strain values. While defect pixels can be interpolated using neighboring pixels or time information, fluctuating strain values are difficult to detect. Fluctuating strain values would have a large impact on the evaluation approach, as the differences between stages are used to detect the onset of necking. This effect can be attenuated to a certain degree using quantile normalization, however, outliers are still introduced as the orientation and magnitude of edges (by extension, the HoG features) are affected. An example of fluctuating pixels or wrong measurements is depicted in Figure 12, with its effects on the difference.

**Figure 13:**
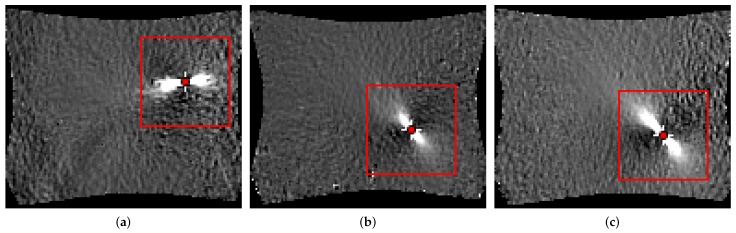
Different orientations of the trials of DP800 - S245-biaxial: (**a**) trial - 1; (**b**) trial - 2; (**c**) trial - 3.

Further consideration is required for the geometry S245 under biaxial straining. The symmetry of the strain condition as well as of the geometry does not permit to assign a preference in the direction and thus the crack can occur in arbitrary direction. On the one hand, the placement of the test S245 could be help. In fact, it is supposed, that the crack tends to occur in the weakest direction, in general perpendicular to the rolling direction for steels and parallel to the rolling direction for aluminum alloys. On the other hand, due to the material anisotropy as well as material structure defects, the onset of crack can still happen independently to the rolling direction. This has no effect on the result of the standard or “line-fit” method as they do not evaluate the gradient with respect to the orientation. In this study the HoG feature is used and as it is not rotation invariant, the orientation of specimen placement seems important, as the gradient develops with specific orientation during forming. This behavior is described in Figure 13 based on the last valid stage of the necking region. Two trials have corresponding or slightly varying necking orientations, while one trial clearly deviates.

**Figure 14:**
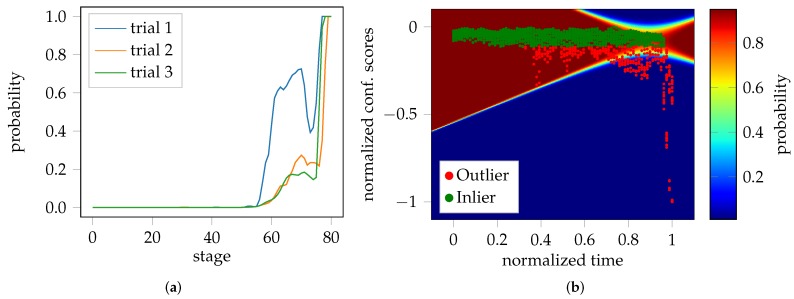
Measurement noise, defect pixel or sample orientation may affect the probability progression in case of S245 geometry: (**a**) Probability progression of DP800-S245 with HoG 180–10° feature; (**b**) Corresponding distribution and decision boundary of the inlier class. Two of the three trials show good agreement while trial 1 negatively affects the inlier and outlier distribution.

However, the orientation during training is addressed using augmentation with random rotations and flipping of the data. To address this, one improvement for the biaxial loading condition might be pose normalization with respect to the last valid stage. The effect of these factors on the probability progression and decision boundary is depicted in Figure 14 using an insufficient HoG resolution of 180° and 10° steps. Trial 1 deviates from trial 2 and 3, that lead to the outliers in the decision boundary visible in Figure 14b and affect the quantile-based strain values. However, the spontaneous failure of this geometry is still consistently captured at ≈76 stage of the test set of each trial, as highlighted by the steep increase in the probability progression curves.

## 5. Conclusions

Previous studies have shown that Nakajima-based forming processes induce visible patterns on the surface of sheet metal materials. In our previous study, presented in Part 1, video sequences and thus images containing these patterns were divided into multiple failure categories by experts. Based on these experts’ annotations a classifier was trained to automatically identify images of unseen video sequences as belonging to one of three different failure classes. However, this evaluation strategy requires expert knowledge and expensive data annotation to facilitate the training of a supervised classification algorithm possible. For this reason, the present study introduces an unsupervised classification method that automatically detects the onset of localized necking without the need for annotated data. Two evaluation methods were presented: (1) a deterministic FLC, which defines an lower and upper bound for the onset of localized necking; (2) a probabilistic FLC, which supports generation of probability quantiles to assess the likelihood of being in the necking phase.

Despite the encouraging results achieved in this study, the proposed method still has several limitations: (1) it is feature dependent, which means that the method only works if some increasing gradient or localization effect is observable; (2) the evaluation of the biaxial S245 geometry is rotation dependent, which may be addressed by pose normalization or through the use of an extended augmentation scheme; (3) the point in time can be well determined based on anomaly detection, however, the choice of evaluation area to obtain the strain values used in the FLC is a heuristic and should be chosen automatically with respect to the investigated material and its geometry. Especially for S100-S125, the evaluation area should flexibly be adapted, as the necking region appears extended; (4) specimen preparation is critical as measurement errors or defect pixels may influence the result as the quantile normalized time derivative of the video sequences are used. In the future, feature dependence, lack of robustness due to rotation dependence in the case of S245 and the susceptibility to measurement noise and defect pixel might be reduced using deep learning techniques, which additionally may enable automatic segmentation of the evaluation area. Despite these limitations, the presented work highlights the potential of an unsupervised classification algorithm to determine the onset of necking using gradient-based features. It introduces the deterministic and probabilistic FLC, which are independent of the evaluation area. The latter, in particular, enables the necking phase to be interpreted in terms of quantiles of certainty, introducing new possibilities for risk and process management. 

## Figures and Tables

**Table 1:** Material properties of the investigated materials.

Material	t_0_ (mm)	n	YS (MPa)	TS (MPa)	UE (%)	r_0_	r_90_
DX54D	0.75, 2.00	0.23	164–170	297–322	22–23	1.80	2.22
DP800	1.00	0.16	465	775–797	14–16	0.76	0.90
AC170	1.00	0.24	140–143	244–239	21–23	0.69	0.67

**Table 2:** Database per material with process parameters.

Material (Thickness mm)	# Images	Frequency (Hz)	Punch Velocity (mm/s)	Available Geometries
DX54D (2.00 mm)	80	20	1.5	S030, S060, S080, S100, S125, S245
AC170	60	15	1.0	S050, S060, S080, S100, S110, S125, S245
DP800	160	40	1.0	S050, S060, S110, S125, S245
DX54D (0.75 mm)	160	40	1.0	2S050, S060, S070, S080, S090, S100, S110, S125, S125, S245
